# Preoperative Psychological Competitive Ability Is Associated With Emotional States Six Months After Anterior Cruciate Ligament Reconstruction With Hamstring Autograft: A Prospective Study

**DOI:** 10.7759/cureus.69099

**Published:** 2024-09-10

**Authors:** Takuya Sengoku, Yasushi Takata, Rikuto Yoshimizu, Mitsuhiro Kimura, Tomoyuki Kanayama, Katsuhiko Kitaoka, Junsuke Nakase

**Affiliations:** 1 Section of Rehabilitation, Kanazawa University Hospital, Kanazawa, JPN; 2 Department of Orthopedic Surgery, Graduate School of Medical Sciences, Kanazawa University, Kanazawa, JPN; 3 Department of Orthopedic Surgery, Kijima Hospital, Kanazawa, JPN

**Keywords:** anterior cruciate ligament (acl), emotion, preoperative psychology, psychological competitive ability, return to practice

## Abstract

Background

Psychological state has been reported as one of the factors strongly related to a return to sports after anterior cruciate ligament (ACL) reconstruction. However, its relationship with the emotional state remains unclear. The aim of this study was to investigate whether patients who have undergone ACL reconstruction and have a higher preoperative psychological competitive ability have a better emotional status preoperatively and six months postoperatively.

Methods

Patients with a Tegner activity score of ≥6 who underwent ACL reconstruction between 2015 and 2020 were divided into two groups according to their grades on the Diagnostic Inventory of Psychological Competitive Ability for Athletes (DIPCA.3). The emotional states preoperatively and at six months postoperatively were assessed using the Profile of Mood States Second Edition (POMS2) and compared between the two groups. Furthermore, the possibility of returning to sports was compared between the groups based on participation in the entire practice at six months postoperatively.

Results

Eighty-four patients were included and divided into the high (DIPCA.3 grades ≥4, n = 23) and low (DIPCA.3 <4, n = 61) groups. Vigor-activity and friendliness were significantly higher in the high group than in the low group preoperatively. The difference was even greater at six months after ACL reconstruction. In addition, the high group showed significantly better results postoperatively for fatigue-inertia and total mood disturbance. Rates of return to sports did not differ significantly between the high and low groups (56.5% vs. 54.1%).

Conclusions

Those with a higher preoperative psychological competitive ability were in a positive emotional state preoperatively and six months after ACL reconstruction. However, the psychological competitive ability did not affect the rate of participation in the entire practice at six months postoperatively.

## Introduction

Anterior cruciate ligament (ACL) injury is a frequent knee trauma in sports [1]. Surgery is the first choice of treatment to regain stable knee joint function and enable a return to sports. Patients often have high expectations that ACL reconstruction will result in good knee function and allow a return to the pre-injury level of sports [2]. However, a large-scale study by Ardern et al. [3] revealed that the rate of returning to sports at a competitive level after ACL reconstruction is low, with 81%, 65%, and 55% returning to sports at any level, preoperative level, and competitive level, respectively. These results indicate the difficulty of returning to sports at a competitive level. Previous studies have identified many factors associated with a return to sports following ACL reconstruction, including age [4], sex [4], knee joint function [5], level of competition [3,4], and psychological state [5,6]. In recent years, several reports have concluded that psychological state is the most relevant factor in the return to sports [7,8].

The Diagnostic Inventory of Psychological Competitive Ability for Athletes (DIPCA.3) is a questionnaire that allows assessment of the psychological aspects of competitive ability. Although there are only a few reports on the use of DIPCA.3 in patients undergoing ACL reconstruction [9,10], a high psychological competitive ability has been reported to be associated with an ACL injury [9]. In recent studies, fear has been widely reported as a negative factor involved in the psychological state of patients returning to sports after ACL reconstruction [11,12]. It is well known that the functional outcome of patients after ACL reconstruction has a considerable impact on their behavioral, cognitive, and emotional status [13]. Although psychological competitive ability reflects psychological competence in a game situation, it does not provide information on perioperative emotional states. As such, it has been hypothesized that the psychological competitive ability and emotional state may be related to a subsequent return to sports.

The aim of this study was to determine whether the preoperative psychological competitive ability of patients who underwent ACL reconstruction was related to their preoperative and six-month postoperative emotional states. The hypothesis of this study was that patients with higher preoperative psychological competitive ability would exhibit more positive emotional states before and after ACL reconstruction.

This article was previously posted to the Research Square preprint server on May 17, 2023 [14].

## Materials and methods

Participants

This study was conducted at Kanazawa University, Kanazawa, Japan, in accordance with the ethical standards laid down in the 1964 Declaration of Helsinki and its later amendments, and was approved by the Ethics Review Committee of Kanazawa University (approval number 2015-058(1860)). Written informed consent was obtained from all patients after the purpose, methods, and potential risks of this study had been fully explained. For patients under 20 years of age, parental consent was obtained.

**Figure 1:**
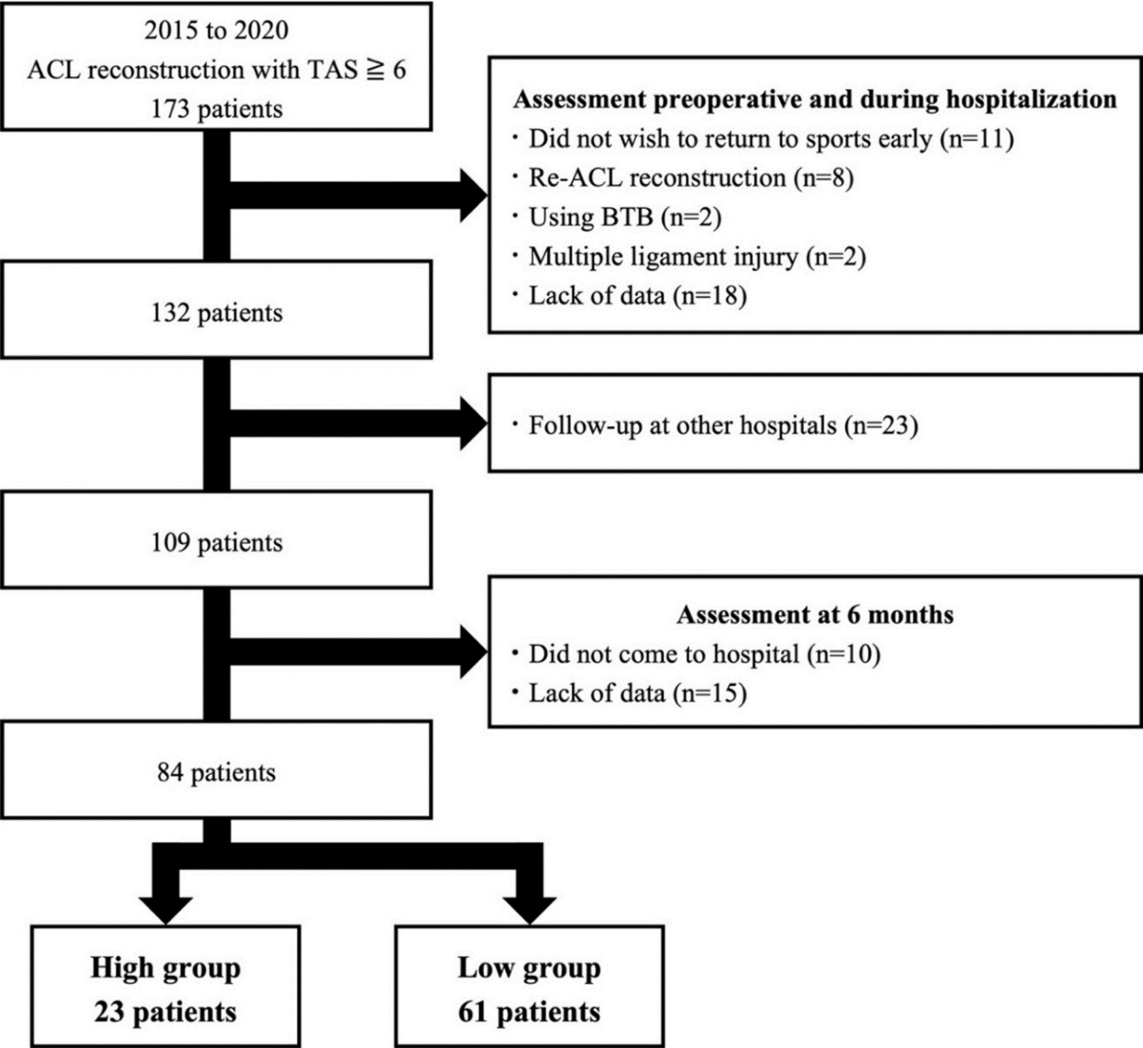
Flowchart of patient selection A total of 173 patients with a Tegner score of ≥6 who underwent ACL reconstruction between 2015 and 2020 were included in this study. Overall, 89 patients were excluded from the preoperative, inpatient, follow-up, or six-month postoperative assessments. Finally, 23 patients in the high group and 61 patients in the low group were included. ACL: anterior cruciate ligament; BTB: bone-patellar tendon-bone; TAS: Tegner activity scale

This prospective study included 173 patients with a Tegner activity scale (TAS) score of 6 or higher who underwent ACL reconstruction at our hospital between 2015 and 2020. The inclusion criteria were as follows: primary ACL reconstruction, hamstring autograft, TAS score ≥6, and availability of data from psychological evaluations conducted preoperatively and at six months after ACL reconstruction. The exclusion criteria were as follows: revision ACL reconstruction, multiple ligament injuries, and incomplete psychological evaluation. The patient selection process is presented in Figure 1.

Psychological evaluation and return to practice

Two questionnaires for psychological assessment were used in this study, the DIPCA.3, which assesses psychological competitive ability, and the Profile of Mood States Second Edition (POMS2), which assesses mood and emotional states. Both questionnaires were applied preoperatively, and POMS2 was further performed six months after ACL reconstruction. Preoperative psychological assessments were performed at the time of hospitalization for ACL reconstruction. A standardized explanation of the questionnaire was provided to all patients in accordance with the instructions.

The DIPCA.3 comprises five main items: volition for competition, mental stability and concentration, confidence, strategic ability, and cooperativeness, which are further subdivided into a total of 12 subitems. The final score is calculated on a scale of 20-240 points, with each item rated on a 5-point scale, with higher scores indicating a better psychological competitive ability (Appendix A) [15].

The POMS2 comprises the following five negative factors: anger-hostility (AH), confusion-bewilderment (CB), depression-dejection (DD), fatigue-inertia (FI), and tension-anxiety (TA). It also comprises the following two positive factors: vigor-activity (VA) and friendliness (F). The five negative factors, subtracted from VA, were calculated as the total mood disturbance (TMD), and F was evaluated independently of the other items. All scores were T-scored and normalized for sex and age. The T-score was assessed on a 50-point threshold, with <50 points indicating a negative factor and ≥50 points indicating a positive factor and a good emotional state (Appendix B).

The rate of return to participation in the entire practice was included in the six-month postoperative evaluation items. In this study, the criterion for returning to sports was defined as participation in the entire practice, with reference to a previous report [16]. Participation in the entire practice in this study excluded game-style practices and referred to other practice programs.

Patients were classified into two groups based on the overall DIPCA.3 grade: high group (grade ≥4) and low group (grade <4). The DIPCA.3 grades were defined as follows: 1, quite low; 2, somewhat low; 3, a bit more; 4, somewhat excellent; and 5, very excellent. The preoperative and six-month postoperative POMS2 scores were compared between the two groups.

Rehabilitation protocol

Postoperative rehabilitation was performed using a program similar to that in a previous study [17]. The preoperative goal was to obtain a range of motion (ROM) of the knee joint from 0° to 125°. Postoperatively, the patients underwent a phased rehabilitation program with the goal of achieving an active ROM from 0° to 120° in one month postoperatively. In addition, isokinetic contraction strength assessed using an isokinetic dynamometer (BIODEX System 4; BIODEX Company, USA) was used as the criterion for return to running and sports activities. Return to running and sports activities were defined by limb symmetry index of ≥60% and ≥90% at three and six months postoperatively, respectively. No active psychological approach was applied to the patients in this study.

Statistical analysis

All statistical analyses were performed using the JMP 14 software (SAS Institute, Cary, NC, USA). Parametric data were analyzed using the Shapiro-Wilk test and F-test. Student’s t-test or Welch’s t-test was performed to assess normally distributed data, and non-parametric data were analyzed using the Wilcoxon test or Pearson’s chi-square test. Statistical significance was set at α = 0.05.

G*Power 3.1.9.4 (Franz Paul, Kiel, Germany) was used to determine the power of the study. An a priori power analysis for the sample size was performed; a sample of 84 patients was required for an effect size of 0.8, an α level of 0.05, and a power of 0.95. Furthermore, a post hoc power analysis was performed to calculate the power and effect size of VA and F preoperatively and of the FI, VA, TMD, and F six months after ACL reconstruction. At preoperatively, the power was VA (0.60) and F (0.95). At six months after ACL reconstruction, a strong power was observed for FI (0.85), VA (0.99), F (0.99), and TMD (0.90). R version 4.2.1 (The R Foundation for Statistical Computing, Austria) was used to calculate the effect size (r). The r value indicated that 0.1, 0.3, and 0.5 were small, medium, and large effect sizes, respectively.

## Results

A total of 84 patients, including 36 (42.9%) men and 48 (57.1%) women, who met all inclusion criteria were enrolled in this study. The average age was 21.1 ± 8.5 years. The high group comprised 23 patients (27.4%, 11 men and 12 women, mean age: 23.1 ± 8.0 years); the low group comprised 61 patients (72.6%, 25 men and 36 women, mean age: 20.3 ± 8.5 years).

There were no significant differences in basic characteristics, such as age, height, weight, or competition level, between the high and low groups (Table 1).

**Table 1:** Patient characteristics Values are presented as mean ± SD. TAS is reported as median (range). Statistical significance is set at α = 0.05. Items with significant differences are marked with an asterisk (*) for p-values: *(p < 0.05), **(p < 0.01). One patient in each group lacked data for the preoperative period. The percentages of meniscus repair and meniscectomy are shown against the number of meniscus tears, respectively. TAS: Tegner activity scale

Characteristic	High group (n = 23)	Low group (n = 61)	p-value
Sex (male:female)	11:12	25:36:00	0.57
Age (years)	23.1 ± 8.0	20.3 ± 8.5	0.11
Height (cm)	166.2 ± 7.9	165.8 ± 8.9	0.85
Weight (kg)	62.5 ± 14.4	61.8 ± 11.1	0.97
BMI (kg/m^2^)	22.4 ± 3.5	22.4 ± 2.9	0.84
TAS	8 (6-9)	7 (6-9)	0.32
Injury status (contact:noncontact)	6:17	12:49	0.53
Preoperative period	44.0 ± 28.9	60.2 ± 38.0	0.05*
Meniscus tear	10 (43.5%)	32 (52.5%)	0.46
Meniscus repair	10 (100%)	31 (96.9%)	0.57
Meniscectomy	0 (0%)	1 (3.1%)	0.57

**Figure 2:**
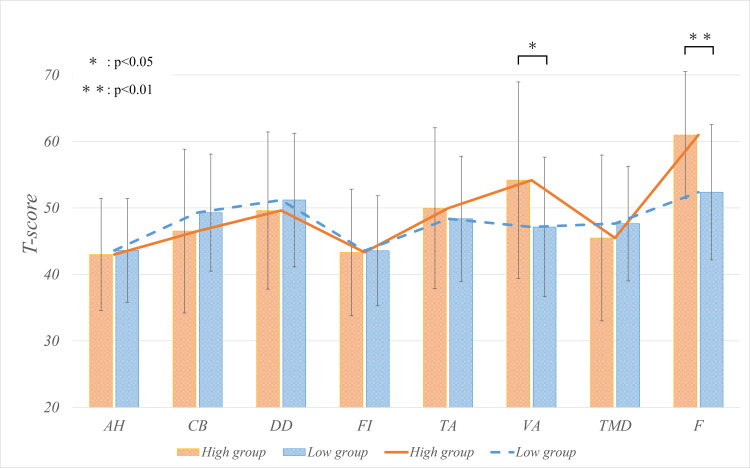
Preoperative POMS2 Statistical significance was set at α = 0.05; a significant intergroup difference was found in the positive items VA and F. AH: anger-hostility; CB: confusion-bewilderment; DD: depress-dejection; F: friendliness; FI: fatigue-inertia; POMS2: Profile of Mood States Second Edition; TA: tension-anxiety; TMD: total mood disturbance; VA: vigor-activity

The preoperative psychological competitive ability scored 201.0 ± 12.7 points in the high group and 155.1 ± 19.5 points in the low group (p < 0.01). There was no significant difference in the preoperative negative items. However, a significant difference was found in the positive items VA and F (Table 2, Figure 2).

**Table 2:** Preoperative POMS2 Values are presented as mean ± SD. Statistical significance is set at α = 0.05. Items with significant differences are marked with an asterisk (*) for p-values: *(p < 0.05), **(p < 0.01). AH: anger-hostility; CB: confusion-bewilderment; DD: depress-dejection; F: friendliness; FI: fatigue-inertia; POMS2: Profile of Mood States Second Edition; TA: tension-anxiety; TMD: total mood disturbance; VA: vigor-activity

Measure	High group (n = 23)	Low group (n = 61)	p-value	r
AH	43.0 ± 8.3	43.6 ± 7.7	0.64	0.08
CB	46.5 ± 12.0	49.3 ± 8.7	0.1	0.18
DD	49.6 ± 11.5	51.2 ± 10.0	0.24	0.13
FI	43.3 ± 9.3	43.6 ± 8.2	0.67	0.05
TA	50.0 ± 11.8	48.4 ± 9.3	0.52	0.04
VA	54.2 ± 14.5	47.1 ± 10.4	0.02*	0.23
TMD	45.5 ± 12.2	47.6 ± 8.6	0.16	0.15
F	61.0 ± 9.4	52.4 ± 10.1	<0.01**	0.36

**Figure 3:**
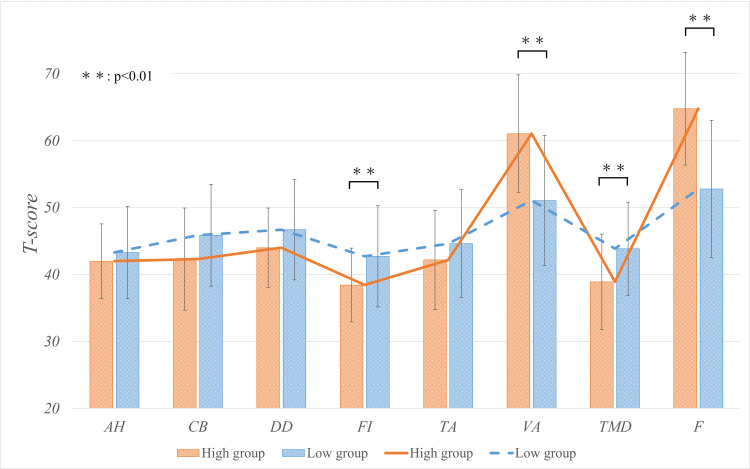
POMS2 at six months after ACL reconstruction Statistical significance was set at α = 0.05. The POMS2 mood profile in the high group showed the iceberg profile, which is exhibited by top athletes. In the iceberg profile, AH, CB, DD, FI, and TA are lower and VA is higher. ACL: anterior cruciate ligament; AH: anger-hostility; CB: confusion-bewilderment, DD: depress-dejection; F: friendliness; FI: fatigue-inertia; POMS2: Profile of Mood States Second Edition; TA: tension-anxiety; TMD: total mood disturbance; VA: vigor-activity

There was also a significant difference in the negative item FI, the positive items VA and F, and in TMD at six months after ACL reconstruction (Table 3, Figure 3).

**Table 3:** POMS2 at six months after ACL reconstruction Values are presented as mean ± SD. Statistical significance is set at α = 0.05. Items with significant differences are marked with an asterisk (*) for p-values: *(p < 0.05), **(p < 0.01). ACL: anterior cruciate ligament; AH: anger-hostility; CB: confusion-bewilderment; DD: depress-dejection; F: friendliness; FI: fatigue-inertia; POMS2: Profile of Mood States Second Edition; TA: tension-anxiety; TMD: total mood disturbance; VA: vigor-activity

Measure	High group (n = 23)	Low group (n = 61)	p-value	r
AH	42.0 ± 5.4	43.3 ± 6.8	0.51	0.07
CB	42.3 ± 7.5	45.9 ± 7.5	0.07	0.2
DD	44.0 ± 5.8	46.7 ± 7.5	0.11	0.18
FI	38.4 ± 5.4	42.7 ± 7.5	<0.01**	0.28
TA	42.2 ± 7.3	44.6 ± 8.0	0.29	0.12
VA	61.0 ± 8.6	51.1 ± 9.7	<0.01**	0.41
TMD	38.9 ± 7.0	43.9 ± 6.9	<0.01**	0.29
F	64.8 ± 8.2	52.8 ± 10.2	<0.01**	0.49

The rate of return to participation in the entire practice at six months after ACL reconstruction was 13 (56.5%) patients in the high group and 33 (54.1%) patients in the low group, with no significant difference between groups.

## Discussion

The most important finding of the present study is that patients with a higher preoperative psychological competitive ability were more likely to show stronger positive emotional states six months after ACL reconstruction. However, the emotional state did not seem to be associated with the rate of returning to participate in the entire practice at six months. Individuals with a higher preoperative psychological competitive ability scored significantly higher in preoperative positive factors, although this study found no significant intergroup difference in the preoperative TMD. At six months after ACL reconstruction, a significant difference in TMD was observed, and the difference in positive factors increased.

The primary goal of ACL reconstruction for athletes at the competitive level is to enable a safe return to sports. Athletes with good psychological and functional status preoperatively have been reported to show good results after ACL reconstruction [18]. In addition, optimism [18] and self-efficacy [19,20] have been reported to contribute to the association between preoperative psychological factors and favorable postoperative outcomes, and negative emotions may have a negative impact on return to sports, daily life situations, and quality of life [21,22]. These findings suggest that positive emotions may have a favorable effect on postoperative outcomes. In this study, the positive items VA and F were found to be significantly higher in the high group than in the low group both preoperatively and at six months after ACL reconstruction. The high group was also found to have significantly higher cooperation in DIPCA.3. In light of these results, the high group was thought to exhibit more positive feelings about interacting with others, which may have had a synergistic effect on vitality and other factors.

Furthermore, the FI was significantly lower and VA was significantly higher in the high group than in the low group, indicating that the high group was more active and energetic and felt less fatigue at six months after ACL reconstruction. As a result, TMD was significantly lower in the high group than in the low group. The mood profile of POMS2 in the high group at six months after ACL reconstruction indicated the iceberg profile reported by Morgan et al. [23], which is thought to be exhibited by top athletes and athletes who have achieved success in sports. The iceberg profile shows low AH, CB, DD, FI, TA, and TMD and high VA, and Figure 3 is a typical iceberg type. A recent meta-analysis [24] concluded that the iceberg profile of POMS2 is an effective indicator for understanding and improving athlete performance. Glazer [25] also reported that psychological readiness to return to sports was negatively correlated with TMD. These results may indicate that patients with a higher preoperative psychological competitive ability have better emotional states six months after ACL reconstruction and are more psychologically ready to return to sports.

However, this study found no significant differences in the rate of returning to participation in the entire practice at six months after ACL reconstruction between the high and low groups. Thus far, no studies have examined the relationship between DIPCA.3 grade and return to sports after ACL reconstruction, so it is difficult to draw conclusions from only this study. Recent reports have recommended that the time to return to sports after ACL reconstruction should be ≥9 months from the perspective of functional improvement and re-injury [20,26]. Based on these considerations, an evaluation at nine months after the surgery is considered necessary.

Limitations

This study has several limitations. First, other factors not considered in this study may be associated with a return to participation in the entire practice and to sports after ACL reconstruction. Further, this study did not investigate the relationship among ROM, muscle strength, and other criteria described in the rehabilitation protocol. Second, no psychological evaluation was conducted over time, and it is unclear how the psychological approach would affect the results of this study. This is a major issue that needs to be addressed in the future, and collaboration with more specialized professionals, such as sports psychologists and clinical psychologists, will be necessary to address this issue. Third, DIPCA.3 was only performed preoperatively and was not assessed six months after ACL reconstruction. Although a psychological approach is required to improve the psychological competitive ability, DIPCA.3 was not used in the assessments at six months postoperatively because no psychological approach was used in this study.

## Conclusions

Those with a higher preoperative psychological competitive ability had better emotional status on positive measures such as preoperative VA and F compared to those with a lower preoperative psychological competitive ability. This difference in emotional state was even more pronounced at six months postoperatively, with those with higher psychological competence showing more positive emotional states. This is an important finding, as it indicates that they are more likely to perform better when performing sports. However, longer-term studies are needed, as no difference was found in return to sport at six months after ACL reconstruction.

## Appendices

Appendix A

**Table 4:** Items and questions of DIPCA.3 DIPCA.3 consists of five main items and twenty sub-items. All questions are scored on a 5-point scale from 1 to 5, with a higher total score meaning the player is more psychologically competitive. Each of the major items is presented on a 5 grade, and the overall DIPCA.3 grade, which is the sum of these items, is likewise presented on a 5 grade. Each of the 5 grades is 1: quite low, 2: somewhat low, 3: a bit more, 4: somewhat excellent, and 5: very excellent. DIPCA.3: Diagnostic Inventory of Psychological Competitive Ability for Athletes

Main items	Sub-items	Questions
Volition for competition	Patience	I can perseveringly compete even in difficult situations.
		I displayed perseverance.
		I can play tough in a game.
		I can sufficiently endure physical pain and exhaustion.
	Aggressiveness	The more major games I’m in the greater fighting spirit I have.
		Whenever there is a game, a fighting spirit begins to stir in me.
		The tougher the opponent, the greater the fight I have.
		Whenever there is an important game, I get all hyped up.
	Volition for self-realization	I compete with attitude of going to the limits of my abilities.
		I go into a game with the thought, “I’ll try hard for myself”.
		I compete with a personal objective in mind.
		I have plenty of personal drive.
	Volition for winning	Before a game, I think “I’m not going to lose"
		Before a game, I think “I want to win by all means"
		Whenever I lose a game, I become a “sore loser”.
		Winning the game, not the essence of the game, is most important to me.
Mental stability and concentration	Self-control	I’m not able to control myself whenever there is a game.
		I’m so nervous I can’t make any of the right moves.
		I am slow to change my feelings.
		My face gets stiff and my hands and legs start shaking.
	Ability to relax	I get nervous worrying too much about winning or losing.
		I get emotionally upset whenever there’s a game.
		I get uneasy before a game.
		I feel pressure whenever there’s a game.
	Concentration	I’m unable to make calm moves.
		I lose my cool at times.
		Whenever there’s a game, the crowds make me upright and I can’t think clearly.
		Thinking about winning or losing makes me uptight and I can’t think clearly.
Confidence	Confidence	I have confidence I can display my abilities even under pressure.
		I have confidence in my personal abilities.
		I have confidence that I will achieve my personal objectives.
		I have confidence I can make my own moves anytime.
	Decision	I can make decisive moves just at the right time.
		I have determination (resolve) in a game.
		I can make decisions without fear of making a mistake.
		I can make quick decisions even in difficult situations.
Strategic ability	Predictive ability	Every strategy of mine proves successful.
		I can change strategy quickly.
		I think of all possible strategies in order to win.
		My predictions are pretty accurate.
	Judgment	I am a person of sound judgment.
		I can judge of the game flow quickly.
		I can make accurate decisions at crucial moments.
		I can make cool decisions even in difficult situations.
Cooperation	Cooperation	I value teamwork.
		During games I and other teammates or partners encourage each other.
		There is a spirit of unity with other teammates.
		During games I and my teammates or partners cooperate well with each other.

Appendix B

**Table 5:** Factors and number of questions for POMS2 The POMS2 is divided into an adult and a youth version (for ages 13-17), both of which consist of seven factors and an overall score. The TMD is calculated as the five negative factors (AH+CB+DD+FI+TA-) - VA. All questions are scored on a 5-point scale from 0-4 and the total score is calculated as a T-score normalized by sex and age. Results are interpreted as a standard score of 50 points, with a score of 50 points or higher being interpreted as good and a score of less than 50 points as poor psychological status. However, the interpretation of the positive items, VA and F, is the opposite. AH: anger-hostility; CB: confusion-bewilderment; DD: depress-dejection; F: friendliness; FI: fatigue-inertia; POMS2: Profile of Mood States Second Edition; TA: tension-anxiety; TMD: total mood disturbance; VA: vigor-activity

Factors	Number of questions (for adults and teenagers)
AH	11/10
CB	10/9
DD	13/8
FI	6/7
TA	10/10
VA	9/11
TMD	－
F	6/5
